# Antibody Responses to *Plasmodium falciparum* and *Plasmodium vivax* and Prospective Risk of *Plasmodium* spp. Infection Postpartum

**DOI:** 10.4269/ajtmh.16-0690

**Published:** 2017-05-03

**Authors:** Alistair R. D. McLean, Machteld Boel, Rose McGready, Ricardo Ataide, Damien Drew, Takafumi Tsuboi, James G. Beeson, François Nosten, Julie A. Simpson, Freya J. I. Fowkes

**Affiliations:** 1 Macfarlane Burnet Institute of Medical Research, Melbourne, Australia; 2 Centre for Epidemiology and Biostatistics, Melbourne School of Population and Global Health, The University of Melbourne, Melbourne, Australia; 3 Shoklo Malaria Research Unit (SMRU), Mahidol-Oxford Tropical Medicine Research Unit, Faculty of Tropical Medicine, Mahidol University, Mae Sot, Thailand; 4 Centre for Tropical Medicine and Global Health, Nuffield Department of Medicine, University of Oxford, Oxford, United Kingdom; 5 Division of Malaria Research, Proteo-Science Center, Ehime University, Ehime, Japan; 6 Department of Microbiology, Monash University, Victoria, Australia; 7 Department of Epidemiology and Preventative Medicine, Monash University, Victoria, Australia

## Abstract

Postpartum women may have an altered susceptibility to *Plasmodium falciparum* and *Plasmodium vivax*. The relationship between naturally acquired malarial immunity and susceptibility to malaria postpartum is yet to be determined. IgG levels were measured against *P. falciparum* and *P. vivax* antigens from delivery in 201 postpartum and 201 nonpregnant controls over 12 weeks. Associations between time-varying antibody levels and time to first microscopically confirmed species-specific infection were determined by Cox regression. Associations between antibody levels and prospective risk of *Plasmodium* infection were similar in postpartum and control women. A 2-fold increase in *P. falciparum* antibody levels was associated with increased prospective risk of *P. falciparum* infection (hazard ratio [HR] range = 1.37–1.94). Antibody levels against most *P. vivax* antigens displayed no association with prospective risk of *P. vivax* infection (HR range = 1.02–1.05) with the exception of *Pv*MSP1_19_ antibodies that were weakly associated with prospective risk of *P. vivax* infection (HR = 1.14 (95% confidence interval = 1.02, 1.28) per 2-fold increase in levels). Associations between antibody levels and prospective risk of infection attenuated when adjusted for documented retrospective exposure. Serology may be a useful tool to predict and monitor women at increased risk of *P. falciparum* infection postpartum, particularly in the absence of a detailed history of retrospective infections.

## Introduction

Pregnant women are at an increased risk of both *Plasmodium falciparum* and *Plasmodium vivax* infections compared with their nonpregnant counterparts.^1^ It is estimated that over 85 million pregnancies each year are at risk of *P. falciparum* infection and 93 million are at risk of *P. vivax* infection.^2^ The increased risk of *P. falciparum* infection has largely been attributed to the ability of the *P. falciparum*-infected erythrocyte (IE) to bind and sequester in the placenta.^3^,^4^ *Plasmodium vivax* does not sequester to the same extent in the placenta, and reasons for altered risk of *P. vivax* infection are less clear, but immunological changes that occur during pregnancy may play a role.^1^ There is emerging evidence that the increased risk during pregnancy may not immediately return to normal after delivery,^5^–^7^ and a recent systematic review demonstrated that postpartum women may be another population at high risk of *Plasmodium* infection and clinical malaria episodes.^8^ The factors responsible for an altered risk of malaria in the postpartum period are unknown.

Individuals living in malaria-endemic regions develop naturally acquired immunity to *P. falciparum* and *P. vivax* with repeated infections. Antibodies are an important component of naturally acquired immunity against malaria.^9^,^10^ Antibodies targeting transmission stages (sporozoites, gametocytes) can prevent liver infection or transmission to mosquitoes and antibodies targeting blood stages (merozoites, IEs) can control parasitemia and prevent the development of clinical symptoms.^11^–^13^ The predominant antigen expressed on the surface of the *P. falciparum* IE is PfEMP1^14^ and a PfEMP1 variant, VAR2CSA, mediates sequestration in the placenta via adherence to chondroitin sulphate A.^3^,^4^ Evidence from population studies suggests that antibody responses of sufficient breadth and magnitude can achieve protection against clinical malaria^15^–^18^ while also acting as biomarkers of past exposure.^19^ Thus, in populations experiencing relatively high homogenous exposure and high levels of immunity, high levels of antibodies against *Plasmodium* spp. have been reported as protective against clinical disease.^20^,^21^ Conversely, in areas where transmission is low and heterogenous, antibodies serve as a marker of increased risk with high-risk exposed individuals generating higher antibody responses compared with individuals with low risk of *Plasmodium* spp. exposure.^22^

Despite extensive literature documenting the increased risk of malaria and *Plasmodium* spp. infection in pregnancy, relatively little is known about the risk of malaria in the postpartum period. There is emerging evidence for an altered susceptibility to *P. falciparum* and *P. vivax* during the postpartum period.^8^ Studies undertaken in Senegal and Gabon reported that postpartum women were at an increased prospective risk of *P. falciparum* infection (relative risk = 1.8 and 2.7, respectively) and clinical falciparum malaria (relative risk = 4.1 and 9.8, respectively) relative to nonpregnant controls.^5^,^6^ Only one study, conducted on the Thailand–Myanmar border, has compared the prospective risk of both *P. falciparum* and *P. vivax* infection in postpartum and nonpregnant women and found that postpartum women experienced significantly less *P. falciparum* infections and significantly more *P. vivax* infections than nonpregnant controls (hazard ratio [HR] = 0.39, 95% confidence interval [CI] = 0.21, 0.72 and HR = 1.34, 95% CI: 1.05, 1.72, respectively).^7^ Despite these epidemiological observations, there have been few immunological investigations on the association of acquired immune responses and risk of infection during the postpartum period.^1^ We previously demonstrated that levels of antibodies against *P. falciparum* and *P. vivax* targets were reduced in postpartum women compared with nonpregnant women, but that these antibody levels recover to normal levels.^23^ The present study sought to investigate the relationship between antibodies specific for *P. falciparum* and *P. vivax* antigens and prospective risk of microscopically confirmed species-specific infection in these postpartum and control (nonpregnant and nonpostpartum) women.^7^

## Materials and Methods

### Ethics statement.

Ethics approval was sought and provided by The Alfred Hospital Human Research and Ethics Committee, Melbourne, Australia (88/13) and the Faculty of Tropical Medicine Ethics Committee, Mahidol University Bangkok, Thailand (MUTM 2007-023) and Oxford Tropical Medicine Ethical Committee, Oxford University, United Kingdom (002-07). All participants gave written, informed, or thumb print, if illiterate, consent before enrollment in the study.

### Study design and population.

This study investigated 201 postpartum women and 201 nonpostpartum women (controls) over a 12-week period. These women represent a subset of women (described previously^23^) from a larger cohort study.^7^ Briefly, pregnant women attending Shoklo Malaria Research Unit (SMRU) antenatal clinics from November 2007 to September 2009 were invited to participate and nonpregnant females of similar age and from same location were recruited as controls. During follow-up, women underwent weekly blood smears and completed questionnaires on behavior. The first serological measurement (baseline) was available at first postpartum visit with additional serological measurements obtained approximately monthly thereafter. Microscopically confirmed *P. falciparum* infections were treated with mefloquine and artesunate. Microscopically confirmed *P. vivax* infections were treated with chloroquine.

### Antibody determination.

Data were available for the levels of total IgG against a variety of *P. falciparum* (*Pf*EBA140_RIII–V_, *Pf*EBA175_RII_, *Pf*EBA175R_III–V_, *Pf*AMA-1, *Pf*MSP2, *Pf*Rh2, *Pf*DBLα, and *Pf*CSP) and *P. vivax* (*Pv*AMA-1, *Pv*MSP1_19_, *Pv*DBP, and *Pv*CSP) antigens as assessed by enzyme-linked immunosorbent assay as well as total IgG binding to the surfaces of *Pf*VAR2CSA-expressing IEs as assessed by flow cytometry.^23^ There were a total of 1,462 serological assessments from 402 women. Seropositivity to a given antigen was defined as an optical density (OD) (or in the case of *Pf*VAR2CSA mean fluorescence intensity [MFI]) greater than the mean + 3 standard deviations derived from 37 negative controls (nonexposed Melbourne donors).

### Statistical analysis.

All statistical analyses were performed using Stata Version 13.1 (StataCorp, College Station, TX). To determine the association between antibody levels and time to first microscopically confirmed *P. falciparum* or *P. vivax* infection, we constructed survival curves comparing high, medium, and low responders (tertiles based on all data) at baseline in postpartum and control subgroups and conducted log-rank tests as a preliminary analysis. Cox proportional hazards regressions were then performed using days after first antibody measurement as time, censoring at the recording of a microscopic *P. falciparum* or *P. vivax* infection; the woman was lost to follow-up; or the woman attended her final visit. Coinfections of *P. vivax* and *P. falciparum* (*N* = 2) were included as a *P. vivax* infection in *P. vivax* survival analyses and a *P. falciparum* infection in *P. falciparum* survival analyses. If a *P. falciparum* infection occurred, the 3-week period after treatment was omitted from the *P. vivax* exposure period, as treatment of *P. falciparum* with mefloquine and artesunate prevents *P. vivax* infection.^24^ If a *P. vivax* infection preceded a *P. falciparum* infection, the analysis was unchanged as chloroquine is not effective against *P. falciparum* in the study area.^25^ Individuals with a *P. vivax* or *P. falciparum* infection on the day of, or before, first antibody measurement but after delivery were excluded from relevant survival analyses. Antibody levels were analyzed as log-transformed continuous variables (log_2_([OD or MFI]+0.001)) or as seropositive/seronegative (in analyses presented in Supplemental Tables 1 and 2).

The assumption of a linear association between antibody levels and the log hazard of infection was tested by comparing Cox regression models with categorical (quartile groupings) and pseudo-continuous antibody variables by likelihood ratio tests. Antibodies were modeled as time-varying exposures, where the most recent measurement was utilized for the following period of exposure. For adjusted analyses, confounders were selected a priori based on a causal diagram^26^ and included clinic visited (Wang Pha/Mawker Tai/Walley/Mu Ler Chai), maternal age (years), use of bednets every night during follow-up (yes/no), slept outside at any time during follow-up (yes/no), and work undertaken outside at any time during follow-up (yes/no).

To determine whether associations between antibody levels and time to first microscopically confirmed infection varied according to postpartum status a likelihood ratio test was performed comparing the model with and without an interaction term for postpartum status (postpartum/control). Where significant interactions were not observed, output from the simpler model was considered for the overall interpretation of results.

## Results

### Characteristics of postpartum and control women.

A total of 201 postpartum women and 201 control women were included in the immunological study. Eleven postpartum women and 12 control women experienced a microscopically confirmed *P. falciparum* infection during the follow-up period after their first antibody measurement. Forty-eight postpartum women and 36 control women experienced a microscopically confirmed *P. vivax* infection in the same period. One postpartum woman and one control woman experienced a coinfection of *P. falciparum* and *P. vivax*. Most individuals with microscopically confirmed infections did not have fever at the time of testing; there were only six febrile *P. falciparum* infections and six febrile *P. vivax* infections. Postpartum women were of a higher gravidity (median of 3 versus 2, *P* < 0.001) and were less likely to sleep and work outside (13.4% versus 19.9%, *P* = 0.08; and 18.4% versus 58.7%, *P* < 0.001) than control women (Table 1).

### *Plasmodium falciparum* antibodies and prospective risk of *P. falciparum* infection in the postpartum period.

**Figure 1.**
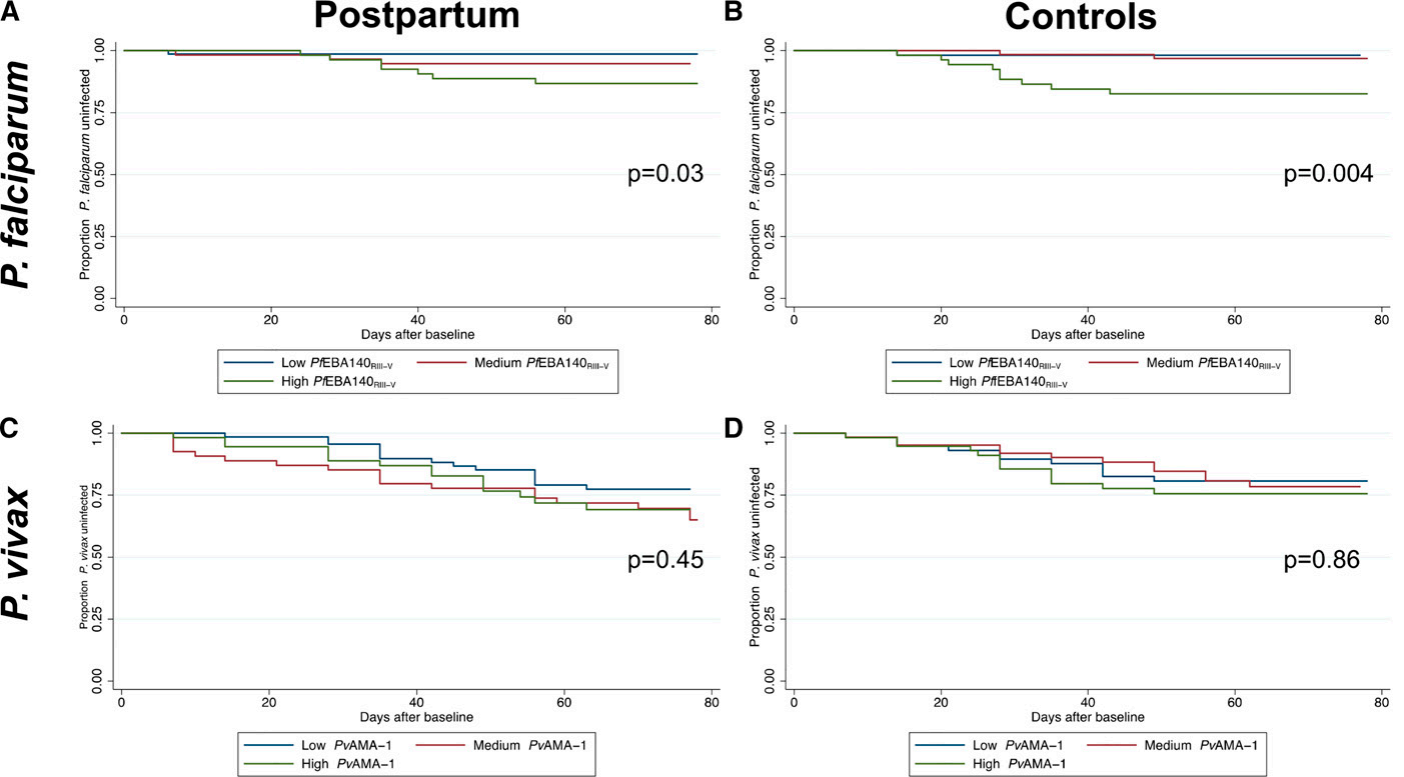
Kaplan–Meier survival curves showing prospective risk of *Plasmodium falciparum* infection among baseline tertiles of *Pf*EBA140_RIII–V_ responders in postpartum (**A**) and controls (**B**) and the prospective risk of *Plasmodium vivax* infection among baseline tertiles of *Pv*AMA-1 responders in postpartum (**C**) and controls (**D**).

Survival curves indicated that women with high antibody responses specific for *P. falciparum* antigens (*Pf*EBA140_RIII–V_, *Pf*EBA175_RII_, *Pf*EBA175R_III–V_, *Pf*AMA-1, *Pf*MSP2, *Pf*Rh2, *Pf*DBLα, *Pf*CSP, and *Pf*VAR2CSA) at baseline had shorter time to first microscopic *P. falciparum* infection compared with those with medium and low antibody responses in both postpartum and control women (*Pf*EBA140_RIII–V_, Figure 1A

, *P* = 0.03 and 1B, *P* = 0.004, respectively; see Supplemental Figures 1 and 2 for other *P. falciparum* antigens).

Univariable and multivariable Cox regression models (adjusting for exposure behaviors, age, and antenatal clinic attended) were used to assess the relationship between antibodies (log_2_(units), time varying) specific for *P. falciparum* antigens with prospective risk of microscopic *P. falciparum* infection in the postpartum period (Table 2). Multivariable analyses showed that antibodies to each *P. falciparum* antigen were associated with an increased adjusted prospective risk of *P. falciparum* infection similarly in postpartum women and controls; for each 2-fold increase in OD there was a 3–79% increase (depending on antigen) in adjusted prospective risk of *P. falciparum* infection in postpartum women and a 14–75% increased adjusted prospective risk in control women (Table 2). The relationship between *P. falciparum* antibodies and prospective risk of infection did not significantly vary between postpartum and control women (likelihood ratio test, all *P* > 0.17). Similar results were observed when comparing seropositive women to seronegative women (Supplemental Table 1). *Plasmodium falciparum* seropositive women had a 2.8- to 17.3-fold (depending on antibody) increase in the prospective risk of *P. falciparum* infection than a woman who was seronegative (*P* < 0.05 for all antibodies). Overall, antibodies specific for *P. falciparum* antigens were associated with prospective risk of infection similarly in postpartum and control women.

### *Plasmodium vivax* antibodies and prospective risk of *P. vivax* infection in the postpartum period.

Survival curves for *P. vivax* antibody responses at baseline showed that high, medium, and low responders against *Pv*CSP, *Pv*DBP, and *Pv*AMA1 tended to have similar times to first microscopically confirmed *P. vivax* infection in postpartum and control women (*Pv*AMA-1, Figure 1C and D, *P* > 0.45; see Supplemental Figure 3 for other *P. vivax* antigens). In contrast, *Pv*MSP1_19_ survival curves suggested that high responders to *Pv*MSP1_19_ had a slightly shorter time to first *P. vivax* infection (*P* = 0.04 for control women and *P* = 0.37 for postpartum women).

Multivariable Cox regression showed that levels (log_2_(OD), time varying) of antibodies specific for *Pv*CSP, *Pv*DBP, and *Pv*AMA1 were not associated with prospective risk of *P. vivax* infection in all (postpartum and control) women (estimated HRs ranged from 0.99 to 1.14; Table 3). However, a 2-fold increase in *Pv*MSP1_19_ levels (OD) was associated with a modest 20% (HR = 1.20, 95% CI = 1.02 to 1.40) and 11% (HR = 1.11, 95% CI = 0.94 to 1.33) increased prospective risk of *P. vivax* infection among postpartum and control women, respectively, after adjustment. The relationship between *P. vivax* antibodies and prospective risk of infection did not significantly vary between postpartum and control women (likelihood ratio test, all *P* > 0.16). Similar results were observed when comparing seropositive women to seronegative women (Supplemental Table 2). In this population, antibodies specific for most *P. vivax* antigens were not associated with *P. vivax* infection in all women.

### Associations between antibody levels and prospective risk of infection attenuate when adjusting for documented retrospective infections.

**Figure 2.**
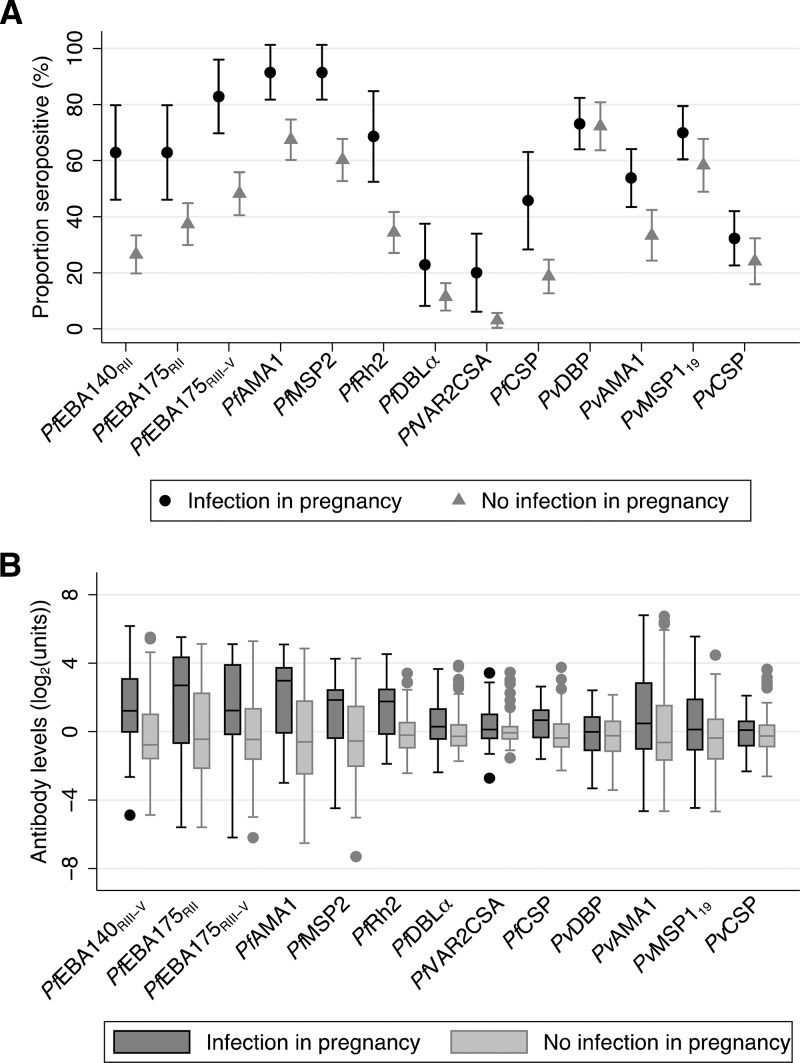
Antibodies to *Plasmodium* species antigens at baseline in postpartum women (*N* = 201) with species-specific infections during pregnancy (*N* = 35 and *N* = 93 for *Plasmodium falciparum* and *Plasmodium vivax* infections, respectively) and without species-specific infections during pregnancy (*N* = 166 and *N* = 108 for absence of *P. falciparum* and *P. vivax* infections, respectively). (**A**) Seroprevalence against *P. falciparum* and *P. vivax* antigens in women with species-specific infection during pregnancy (black circles) and women who did not experience a species-specific infection during pregnancy (gray triangles). Bars indicate 95% confidence intervals. *P* < 0.05 for all antibodies except *Pf*DBLα (*P* = 0.09), *Pv*DBP (*P* = 0.89), *Pv*MSP1_19_ (*P* = 0.09), and *Pv*CSP (*P* = 0.20). (**B**) Box and whiskers plots of IgG levels (log_2_(MFI) for *Pf*VAR2CSA, log_2_(OD) for all other antibodies) against *P. falciparum* and *P. vivax* antigens in women with species-specific infection during pregnancy (black) and women who did not experience a species-specific infection during pregnancy (gray). Horizontal line in box indicates median, box indicates the interquartile range, whiskers indicate the highest and lowest values within 1.5 × interquartile range of the first and third quartiles, dots represent outliers. *P* < 0.05 for all antibodies except *Pf*VAR2CSA (*P* = 0.22), *Pv*DBP (*P* = 0.35), and *Pv*CSP (*P* = 0.14). MFI = mean fluorescence intensity; OD = optical density.

To assess whether antibodies were acting as biomarkers of retrospective exposure, we investigated the association of antibodies specific for *Plasmodium* spp. at baseline with retrospective *Plasmodium* spp. infections in postpartum women who had been screened weekly during pregnancy for the presence of *Plasmodium* spp. parasites. Antibody levels and seroprevalence to *P. falciparum* antigens were higher among postpartum women who experienced a *P. falciparum* infection during pregnancy (*N* = 35) compared with postpartum women who remained *P. falciparum* free during pregnancy (*N* = 166) (Figure 2

). Similarly, postpartum women who experienced a *P. vivax* infection during pregnancy (*N* = 93) tended to have higher levels and seroprevalence of antibodies against *P. vivax* targets than postpartum women who had no *P. vivax* infections during pregnancy (*N* = 108). However, the magnitude of difference in *P. vivax* antibody responses was smaller than that observed for *P. falciparum* antibody responses between pregnancy exposure groups (Figure 2).

Adjusting for a documented *P. falciparum* infection during pregnancy in the postpartum women reduced the magnitude of association between *P. falciparum* antibodies and prospective risk of *P. falciparum* infection (median [minimum–maximum] of all HR estimates unadjusted for history = 1.36 [1.01–1.81], adjusted for history = 1.26 [0.85–1.52]) (Supplemental Table 3). Similarly, adjusting for a documented *P. vivax* infection during pregnancy in postpartum women reduced the magnitude of association between *Pv*MSP1_19_ and prospective risk of *P. vivax* infection (Supplemental Table 3) (HR unadjusted for history = 1.18, 95% CI = 1.00, 1.38; adjusted for history = 1.12, 95% CI = 0.95, 1.32). However, adjusting for retrospective infections as assessed by questionnaire in the control women did not alter the magnitude of associations (Supplemental Table 4). Positive associations between antibodies and prospective risk of *Plasmodium* spp. infection are likely reflective of a subset of women at high risk of *Plasmodium* spp. exposure (both retrospective and prospective).

## Discussion

In the first study investigating humoral immunity and prospective risk of *P. falciparum* and *P. vivax* infection postpartum, all *P. falciparum* antibodies investigated showed a positive association with prospective risk of *P. falciparum* infection, and there was no statistical evidence that this association differed for postpartum and control women. Only *Pv*MSP1_19_ antibodies, but not *Pv*AMA-1, *Pv*DBP, or *Pv*CSP antibodies, showed a positive association with prospective risk of *P. vivax* infection. Associations between antibody levels and prospective risk of infection were broadly comparable in postpartum and control women. In this low-endemic setting, antibodies specific for *P. falciparum*, but not for *P. vivax*, acted as biomarkers of both retrospective and prospective risk of infection, in both postpartum and control women.

Antibodies specific for *P. falciparum* antigens were associated with an increased prospective risk of *P. falciparum* infection in both postpartum and controls. Antibodies specific for *P. falciparum* antigens were considerably higher in postpartum women who had experienced a *P. falciparum* infection in pregnancy compared with those who had not and were therefore indicative of retrospective *P. falciparum* exposure in this population. The association of *P. falciparum* antibodies with prospective risk of infection is in line with studies from other populations in low-transmission settings, which have also reported positive associations between *P. falciparum* antibodies and risk of infection.^21^,^27^,^28^

Unlike *P. falciparum* antibody responses, antibodies specific for *P. vivax* antigens showed no association with prospective risk of *P. vivax*, with the exception of antibodies against *Pv*MSP1_19_. Antibodies against *P. vivax* antigens were typically higher in women who experienced an infection during pregnancy than those who were uninfected but the effect was less pronounced than that observed in response to *P. falciparum* targets. The association of antibodies against *Pv*MSP1_19_, but not other *P. vivax* targets, with prospective risk of *P. vivax* infection suggests that *Pv*MSP1_19_ may be more immunogenic than other *P. vivax* targets. Indeed, our earlier research indicated that antibodies against *Pv*MSP1_19_ were more responsive to *P. vivax* infection than the other antibody responses investigated.^23^ Another key difference between *P. falciparum* and *P. vivax* is that *P. vivax* possesses the ability to form hypnozoites in the liver, a dormant stage that can lead to relapses of blood-stage infections.^29^ Relapses are responsible for a high proportion of observed *P. vivax* infections,^30^,^31^ so any reduction in vector exposure that occurs postpartum will have a negligible impact on prospective risk of a blood-stage *P. vivax* infection. These data suggest that *P. vivax* antibodies against targets investigated in this study have limited utility in detecting retrospective exposure to *P. vivax* or predicting future exposure to *P. vivax* in this population.

Antibodies specific for *P. falciparum* and *P. vivax* were present at higher levels in women who had experienced a retrospective infection. Given that postpartum and control women were monitored for malaria differently in this period (weekly testing by microscopy versus a one-off retrospective questionnaire), we were not able to directly compare the effect of a retrospective infection across all women. Instead we ran subgroup analyses within postpartum and control women. Adjusting for infection during pregnancy reduced the association between antibodies and prospective risk of *Plasmodium* spp. infection in postpartum women, but adjustment for retrospective history of malaria by questionnaire in control women did not alter the magnitude of associations most likely because the questionnaire, unlike microscopy, did not accurately define prior malaria episodes. Our results suggest that serology may be a useful tool to predict and monitor individuals and populations at increased risk of *P. falciparum*, particularly in the absence of a detailed history of retrospective infections.

In the two other studies that have investigated the risk of *P. falciparum* infection in postpartum women compared with nonpregnant control women, an increased risk of infection in the postpartum women was observed (relative risk = 1.8 for *P. falciparum* infection, 4.1 for clinical malaria^5^; and incident rate ratio = 2.7 for *P. falciparum* infection, 9.8 for clinical malaria^6^). These studies, performed in high-transmission areas in Africa, are in stark contrast to unadjusted analyses of the present study population in a low-transmission area of Thailand where postpartum women were at reduced prospective risk of *P. falciparum* infection (HR = 0.39).^7^ The study settings in African and Asia centers differ in key respects. At SMRU, testing for the presence of *Plasmodium* spp. infection by light microscopy occurs weekly, with infected women receiving prompt treatment. This treatment is typically before the commencement of symptomatic malaria, precluding an assessment of clinical immunity. As such, associations investigated in this study were measuring the effect of antibodies on an individual having an infection with parasitemia greater than 50 parasites/μL (detection threshold of light microscopy).^32^ Both studies in Africa were conducted in areas of high *P. falciparum* endemicity^5^,^6^ where asymptomatic *P. falciparum* infections were not treated routinely. They found that, for a given *P. falciparum* infection, postpartum women were more likely to develop clinical symptoms compared with control women.^5^,^6^ Whether impaired humoral immunity in postpartum women reduces their ability to control an infection below a clinical pyrogenic threshold is unknown, but past research in other populations has suggested that even where increased levels of antibodies indicate an increased risk of infection, higher levels of antibodies are associated with a reduced likelihood of developing symptoms given infection.^27^ A transient reduction in antibody levels post pregnancy^23^ may be responsible for an increased risk of infection and disease in the African studies, but these studies did not investigate serology. As such, studies of immunity to malaria in postpartum women are warranted in high-transmission settings.

This study provides a comprehensive analysis of the relationship between antibodies toward two *Plasmodium* spp. and prospective risk of infection postpartum. Antibodies against numerous *P. falciparum* targets acted as biomarkers of exposure; only antibodies specific for *Pv*MSP1_19_ demonstrated any association with prospective risk of *P. vivax* infection. Further studies are required in high-transmission, high-immunity settings, particularly exploring the association between immunity and symptomatic episode endpoints. In addition, given that this is the only population where prospective risk of *P. vivax* postpartum has been investigated, further immunoepidemiological studies of *P. vivax* in other postpartum populations are needed to assess the generalizability of findings. While this study focused on antibody responses, additional research should be conducted into the relationship between antibody-independent mechanisms, such as innate and cell-mediated immunity, and susceptibility to malaria in the postpartum period.

## Supplementary Material

Supplemental Datas.

## Figures and Tables

**Table 1:** Characteristics of postpartum women and control women

	Postpartum (*N* = 201)	Controls (*N* = 201)	*P* value
At enrollment			
Age (years)	27.5 (22–32) [18–45.5]	28 (23–35) [18–50]	0.40
Gravidity			< 0.001
Nulligravid	0 (0)	38 (18.9)	
1–2	78 (38.8)	75 (37.3)	
3+	123 (61.2)	88 (43.8)	
History of malaria*			
*Plasmodium falciparum* in last 9 months	35 (17.4)	10 (5.0)	< 0.001
*Plasmodium vivax* in last 9 months	93 (46.3)	11 (5.5)	< 0.001
During follow-up			
*P. falciparum*†			
Any infection	11 (5.8)	12 (6.7)	0.72
Number of infections	1 (1–2) [1–3]	1 (1–1.5) [1–2]	0.53
*P. vivax*‡			
Any infection	48 (26.3)	36 (19.5)	0.12
Number of infections	1 (1–1) [1–2]	1 (1–1) [1–3]	0.48
Behavior			
Use of bednets	174 (86.6)	184 (91.5)	0.11
Slept outside	27 (13.4)	40 (19.9)	0.08
Worked outside	37 (18.4)	118 (58.7)	< 0.001

Data presented as median (interquartile range) [minimum–maximum] or *n* (%). Wilcoxon rank-sum tests were performed on continuous data; χ^2^ tests were performed on categorical data.

* Malaria history data collected differently between groups: postpartum women had experienced weekly smears during their pregnancy; control women had not been monitored before inclusion.

† Among women included in *P. falciparum* survival analysis (189 postpartum, 178 controls).

‡ Among women included in *P. vivax* survival analysis (182 postpartum, 185 controls).

**Table 2:** Antibodies and prospective risk of *Plasmodium falciparum* infection

Antibody (log_2_(units*))	All women		Postpartum†		Control†		LR test for interaction‡
	HR (95% CI)	*P* value	HR (95% CI)	*P* value	HR (95% CI)	*P* value	*P* value
Unadjusted							
*Pf*EBA140_RIII–V_	1.41 (1.21, 1.65)	< 0.001	1.44 (1.13, 1.83)	0.003	1.41 (1.15, 1.72)	0.001	0.89
*Pf*EBA175_RII_	1.37 (1.15, 1.62)	< 0.001	1.37 (1.06, 1.77)	0.02	1.36 (1.08, 1.70)	0.01	0.95
*Pf*EBA175_RIII–V_	1.52 (1.26, 1.83)	< 0.001	1.44 (1.11, 1.87)	0.007	1.61 (1.23, 2.11)	0.001	0.56
*Pf*AMA1	1.41 (1.17, 1.69)	< 0.001	1.20 (1.01, 1.68)	0.04	1.51 (1.15, 1.99)	0.003	0.43
*Pf*MSP2	1.46 (1.17, 1.81)	0.001	1.42 (1.04, 1.96)	0.03	1.48 (1.10, 2.00)	0.01	0.86
*Pf*Rh2	1.94 (1.50, 2.50)	< 0.001	1.99 (1.33, 3.00)	0.001	1.91 (1.37, 2.68)	< 0.001	0.88
*Pf*DBLα	1.45 (1.07, 1.95)	0.02	1.12 (0.68, 1.83)	0.66	1.78 (1.18, 2.68)	0.006	0.14
*Pf*VAR2CSA	1.88 (1.36, 2.59)	< 0.001	2.14 (1.41, 3.27)	< 0.001	1.58 (0.93, 2.67)	0.09	0.36
*Pf*CSP	1.57 (1.11, 2.21)	0.01	1.75 (1.09, 2.81)	0.02	1.38 (0.84, 2.27)	0.20	0.50
Adjusted§							
*Pf*EBA140_RIII–V_	1.33 (1.15, 1.54)	< 0.001	1.32 (1.05, 1.65)	0.02	1.35 (1.07, 1.65)	0.003	0.87
*Pf*EBA175_RII_	1.29 (1.10, 1.51)	0.001	1.26 (0.99, 1.60)	0.06	1.32 (1.06, 1.63)	0.01	0.79
*Pf*EBA175_RIII–V_	1.46 (1.21, 1.76)	< 0.001	1.36 (1.03, 1.80)	0.03	1.55 (1.18, 2.03)	0.002	0.53
*Pf*AMA1	1.34 (1.12, 1.61)	0.001	1.22 (0.95, 1.57)	0.12	1.47 (1.13, 1.92)	0.01	0.32
*Pf*MSP2	1.37 (1.10, 1.71)	0.01	1.36 (0.97, 1.89)	0.07	1.39 (1.04, 1.86)	0.03	0.92
*Pf*Rh2	1.67 (1.29, 2.16)	< 0.001	1.54 (1.03, 2.32)	0.04	1.75 (1.25, 2.44)	0.001	0.64
*Pf*DBLα	1.37 (1.03, 1.83)	0.03	1.03 (0.61, 1.76)	0.90	1.63 (1.11, 2.40)	0.01	0.17
*Pf*VAR2CSA	1.88 (1.29, 2.74)	0.001	1.96 (1.21, 3.17)	0.01	1.77 (0.97, 3.20)	0.06	0.78
*Pf*CSP	1.45 (0.97, 2.16)	0.07	1.79 (1.06, 3.02)	0.03	1.14 (0.64, 2.04)	0.65	0.26

CI = confidence interval; HR = hazard ratio; LR = likelihood ratio.

* Mean fluorescence intensity for *Pf*VAR2CSA, optical density for all other antibody measurements.

† Model with interaction term between antibody level and postpartum status.

‡ LR test (*P* value) comparing model with interaction term between antibody level and postpartum status to model without interaction term.

§ Adjusted for exposure behaviors, age, postpartum status, and clinic attended.

**Table 3:** Antibodies and prospective risk of *Plasmodium vivax* infection

Antibody (log_2_(units*))	All women		Postpartum†		Control†		LR test for interaction‡
	HR (95% CI)	*P* value	HR (95% CI)	*P* value	HR (95% CI)	*P* value	*P* value
Unadjusted							
*Pv*DBP	1.02 (0.86, 1.21)	0.82	1.05 (0.84, 1.33)	0.76	1.00 (0.76, 1.31)	0.99	0.76
*Pv*AMA-1	1.05 (0.97, 1.13)	0.23	1.09 (0.99, 1.21)	0.07	0.99 (0.87, 1.12)	0.84	0.20
*Pv*MSP1_19_	1.14 (1.02, 1.28)	0.02	1.17 (1.01, 1.36)	0.03	1.12 (0.95, 1.33)	0.18	0.70
*Pv*CSP	1.04 (0.87, 1.24)	0.67	1.06 (0.84, 1.33)	0.63	1.06 (0.80, 1.37)	0.72	0.98
Adjusted§							
*Pv*DBP	1.03 (0.87, 1.23)	0.71	1.07 (0.85, 1.35)	0.56	0.99 (0.76, 1.29)	0.92	0.65
*Pv*AMA-1	1.06 (0.98, 1.15)	0.15	1.12 (1.01, 1.24)	0.04	0.99 (0.87, 1.12)	0.85	0.12
*Pv*MSP1_19_	1.16 (1.03, 1.30)	0.01	1.20 (1.02, 1.40)	0.03	1.11 (0.94, 1.33)	0.22	0.55
*Pv*CSP	1.11 (0.92, 1.33)	0.28	1.14 (0.89, 1.46)	0.31	1.07 (0.82, 1.40)	0.62	0.75

CI = confidence interval; HR = hazard ratio; LR = likelihood ratio.

* Mean fluorescence intensity for *Pf*VAR2CSA, optical density for all other antibody measurements.

† Model with interaction term between antibody level and postpartum status.

‡ LR test (*P* value) comparing model with interaction term between antibody level and postpartum status to model without interaction term.

§ Adjusted for exposure behaviors, age, postpartum status, and clinic attended.
