# Supplementary material for: Antibody Responses to Plasmodium falciparum and Plasmodium vivax and Prospective Risk of Plasmodium spp. Infection Postpartum
Source: Am J Trop Med Hyg. 2017 May 3;96(5):1197–204. doi: 10.4269/ajtmh.16-0690 (PMC5417217; doi:10.4269/ajtmh.16-0690)
Supplement: Supplementary file 1 [file SD7.pdf]

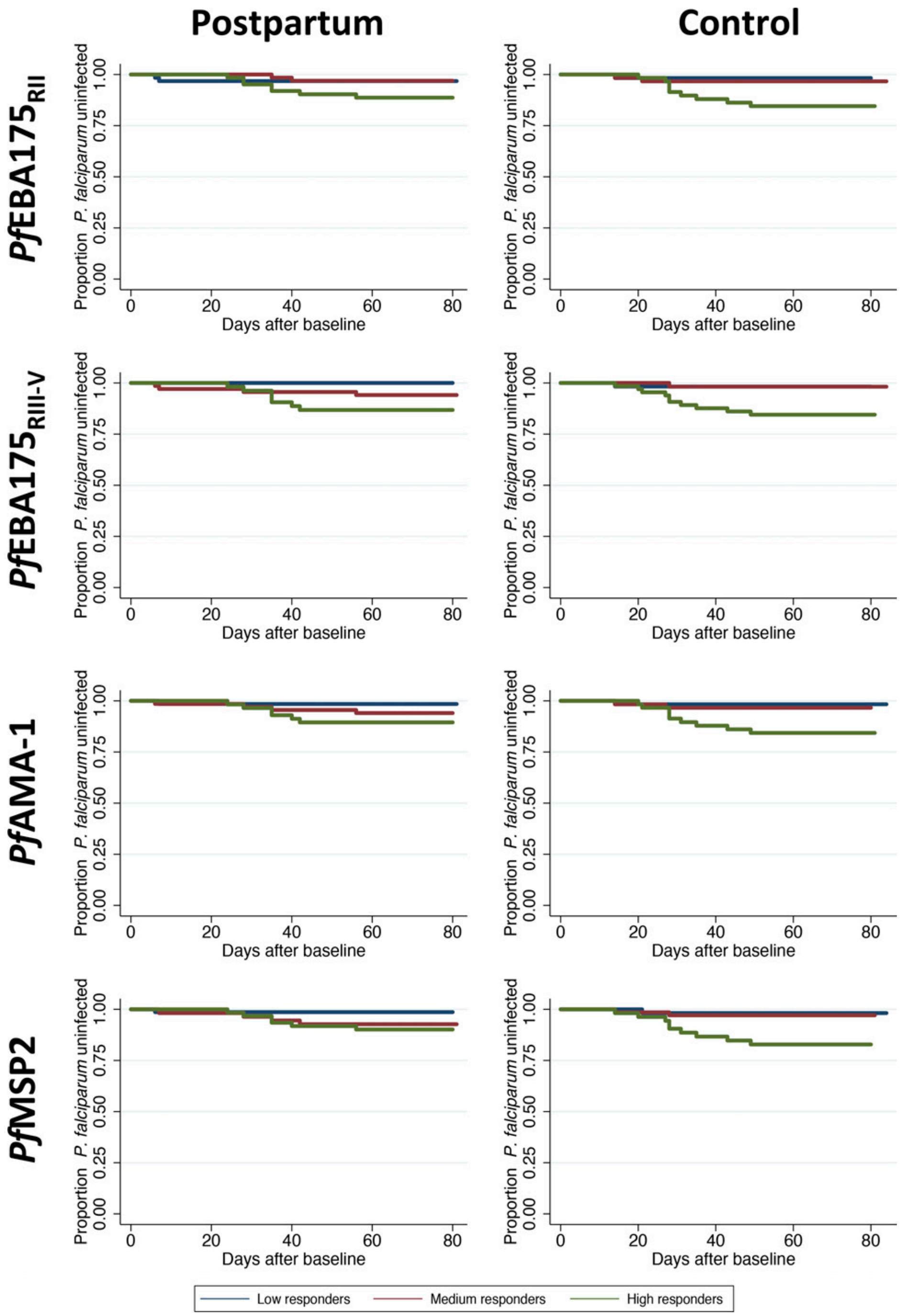

Supplemental Figure 1. Kaplan–Meier curves for *Pf*EBA175$_{RII}$, *Pf*EBA175$_{RIII–V}$, *Pf*AMA1, and *Pf*MSP2 in postpartum and control women. Kaplan–Meier survival curves showing prospective risk of *Plasmodium falciparum* infection among low, medium, and high responders to *Pf*EBA175$_{RII}$, *Pf*EBA175$_{RIII–V}$, *Pf*AMA1, and *Pf*MSP2 for postpartum and control women separately.

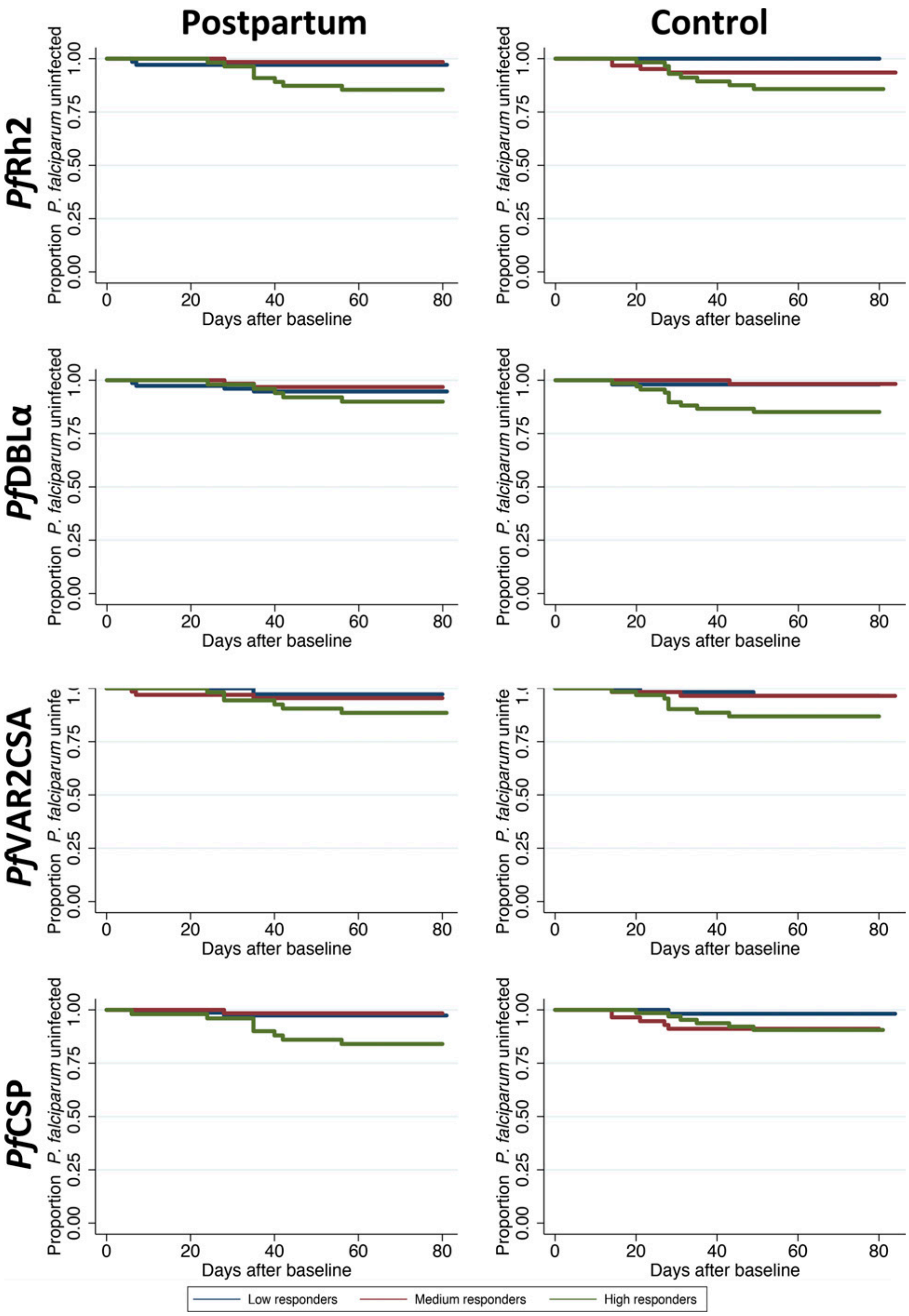

Supplemental Figure 2. Kaplan–Meier curves for *Pf*Rh2, *Pf*DBLα, *Pf*VAR2CSA, and *Pf*CSP in postpartum and control women. Kaplan–Meier survival curves showing prospective risk of *Plasmodium falciparum* infection among low, medium, and high responders to *Pf*Rh2, *Pf*DBLα, *Pf*VAR2CSA, and *Pf*CSP for postpartum and control women separately.

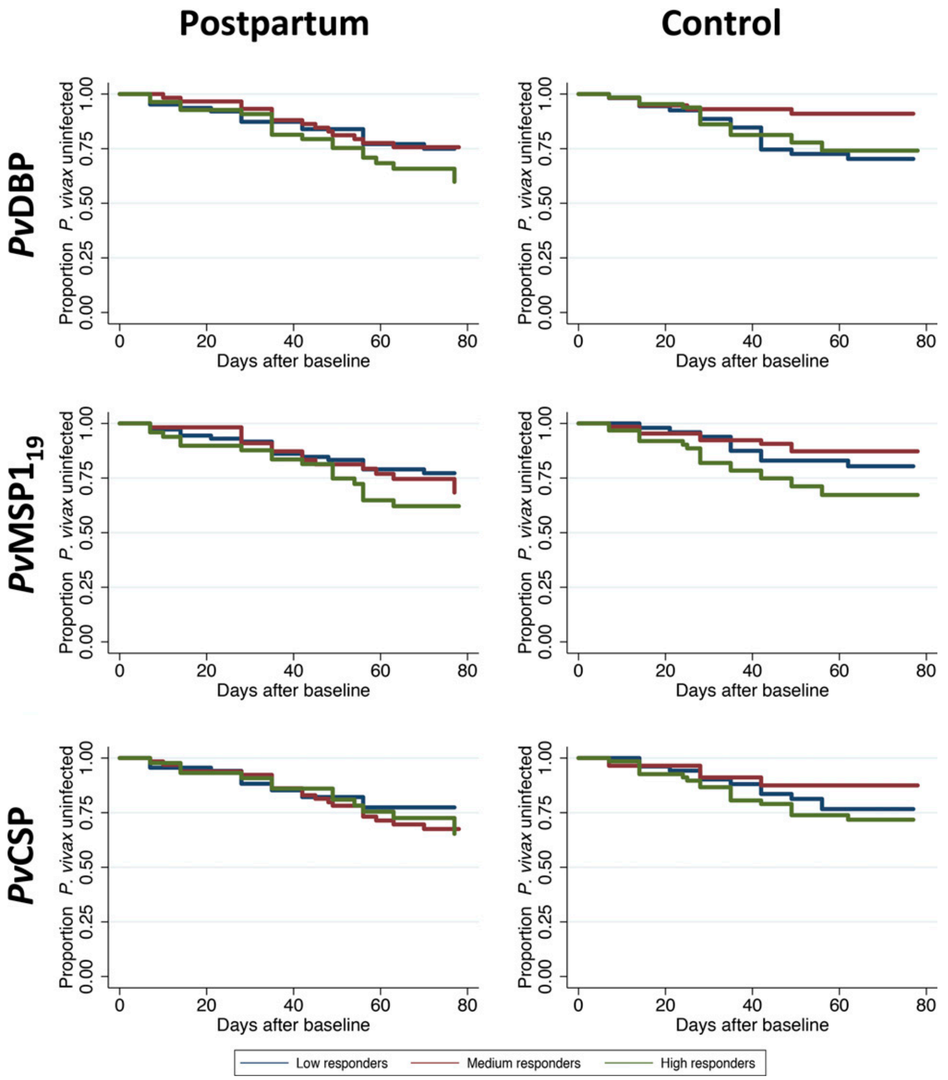

Supplemental Figure 3. Kaplan–Meier curves for *Pv*DBP, *Pv*MSP1_{19}, and *Pv*CSP in postpartum and control women. Kaplan–Meier survival curves showing prospective risk of *Plasmodium vivax* infection among low, medium, and high responders to *Pv*DBP, *Pv*MSP119, and *Pv*CSP for postpartum and control women separately.

Supplemental Table 1

Seroprevalence at baseline and prospective risk of *Plasmodium falciparum* infection

Antibody	Hazard ratio (95% confidence interval); *P* value
*Pf*EBA$140_{RIII–V}$	4.44 (1.82, 10.8); 0.001
*Pf*EBA175_{RII}	4.85 (1.80, 13.1); 0.002
*Pf*EBA$175_{RIII–V}$	17.3 (2.34, 129); 0.01
*Pf*AMA1	4.63 (1.09, 19.8); 0.04
*Pf*MSP2	5.14 (1.20, 21.91); 0.03
*Pf*Rh2	5.33 (1.98, 14.3); 0.001
*Pf*DBLα	2.97 (1.26, 7.01); 0.01
*Pf*VAR2CSA	5.75 (2.26, 14.6); < 0.001
*Pf*CSP	2.77 (1.22, 6.28); 0.02

Supplemental Table 2

Seroprevalence at baseline and prospective risk of *Plasmodium vivax* infection

Antibody	Hazard ratio (95% confidence interval); *P* value
*Pv*DBP	0.84 (0.53, 1.35); 0.48
*Pv*AMA-1	1.37 (0.89, 2.11); 0.15
*Pv*MSP1_{19}	1.44 (0.88, 2.37); 0.15
*Pv*CSP	1.45 (0.93, 2.24); 0.10

Supplemental Table 3

Antibodies and adjusted prospective risk of species-specific infection with and without adjustment for species-specific infection in the last 9 months in postpartum women

	Hazard ratio (95% confidence interval); *P* value	
Antibody (log_2(units*))	Adjusted for confounders†	Adjusted for confounders (history included)‡
Outcome (*Plasmodium falciparum*)		
*Pf*EBA140$_{RIII–V}$	1.36 (1.07, 1.72); 0.01	1.27 (0.96, 1.67); 0.09
*Pf*EBA175$_{RII}$	1.24 (0.98, 1.57); 0.07	1.13 (0.86, 1.48); 0.39
*Pf*EBA175$_{RIII–V}$	1.38 (1.02, 1.86); 0.04	1.26 (0.90, 1.76); 0.18
*Pf*AMA1	1.24 (0.96, 1.59); 0.09	1.10 (0.82, 1.48); 0.51
*Pf*MSP2	1.27 (0.91, 1.78); 0.16	1.12 (0.78, 1.61); 0.55
*Pf*Rh2	1.53 (0.99, 2.37); 0.06	1.27 (0.77, 2.09); 0.35
*Pf*VAR2CSA	2.02 (1.16, 3.50); 0.01	1.92 (1.08, 3.39); 0.03
*Pf*DBLα	1.01 (0.60, 1.70); 0.96	0.85 (0.47, 1.53); 0.58
*Pf*CSP	1.81 (1.00, 3.27); 0.05	1.52 (0.79, 2.90); 0.21
Outcome (*Plasmodium vivax*)		
*Pv*DBP	1.03 (0.82, 1.29); 0.81	1.05 (0.85, 1.31); 0.64
*Pv*AMA-1	1.09 (0.98, 1.21); 0.11	1.09 (0.97, 1.21); 0.13
*Pv*MSP1$_{19}$	1.18 (1.00, 1.38); 0.05	1.12 (0.95, 1.32); 0.18
*Pv*CSP	1.08 (0.84, 1.38); 0.55	1.12 (0.87, 1.45); 0.40

*Mean fluorescence intensity for *Pf*VAR2CSA, optical density for all other antibody measurements.

†Exposure behaviors, age, and antenatal clinic attended.

‡History of infection in postpartum women utilized records in pregnancy, a period when they were screened for infection regularly.

Supplemental Table 4

Antibodies and adjusted prospective risk of species-specific infection with and without adjustment for species-specific infection in the last 9 months in control women

	Hazard ratio (95% confidence interval); *P* value	
Antibody (log_2(units*))	Adjusted for confounders†	Adjusted for confounders (history included)‡
Outcome (*Plasmodium falciparum*)		
*Pf*EBA140$_{RIII–V}$	1.38 (1.11, 1.72); 0.003	1.40 (1.12, 1.76); 0.003
*Pf*EBA175$_{RII}$	1.33 (1.07, 1.67); 0.01	1.33 (1.07, 1.67); 0.01
*Pf*EBA175$_{RIII–V}$	1.55 (1.17, 2.05); 0.002	1.58 (1.18, 2.11); 0.002
*Pf*AMA1	1.46 (1.11, 1.92); 0.01	1.46 (1.11, 1.92); 0.01
*Pf*MSP2	1.38 (1.02, 1.87); 0.04	1.43 (1.04, 1.98); 0.03
*Pf*Rh2	1.79 (1.27, 2.53); 0.001	1.83 (1.28, 2.60); 0.001
*Pf*VAR2CSA	1.75 (0.97, 3.16); 0.06	1.72 (0.94, 3.15); 0.08
*Pf*DBLα	1.60 (1.06, 2.41); 0.02	1.62 (1.08, 2.42); 0.02
*Pf*CSP	1.23 (0.69, 2.19); 0.48	1.23 (0.69, 2.19); 0.48
Outcome (*Plasmodium vivax*)		
*Pv*DBP	1.01 (0.77, 1.33); 0.94	1.01 (0.77, 1.33); 0.95
*Pv*AMA-1	1.00 (0.88, 1.14); 0.97	1.00 (0.88, 1.14); 0.97
*Pv*MSP1$_{19}$	1.16 (0.97, 1.39); 0.10	1.17 (0.97, 1.40); 0.10
*Pv*CSP	1.09 (0.82, 1.45); 0.54	1.09 (0.82, 1.45); 0.55

*Mean fluorescence intensity for *Pf*VAR2CSA, optical density for all other antibody measurements.

†Exposure behaviors, age, and antenatal clinic attended.

‡History of infection in control women was ascertained by retrospective questionnaire and clinic records where available.
